# Obesity, Insulin Resistance, and Hyperandrogenism Mediate the Link between Poor Diet Quality and Ovarian Dysmorphology in Reproductive-Aged Women

**DOI:** 10.3390/nu12071953

**Published:** 2020-06-30

**Authors:** Maryam Kazemi, Brittany Y. Jarrett, Heidi Vanden Brink, Annie W. Lin, Kathleen M. Hoeger, Steven D. Spandorfer, Marla E. Lujan

**Affiliations:** 1 Division of Nutritional Sciences, Cornell University, Ithaca, NY 14853, USA; maryam.kazemi@cornell.edu (M.K.); BYJ4@cornell.edu (B.Y.J.); hv63@cornell.edu (H.V.B.); 2 Department of Preventative Medicine, Feinberg School of Medicine, Northwestern University, Evanston, IL 60611, USA; annie.lin@northwestern.edu; 3 Department of Obstetrics and Gynecology, University of Rochester Medical Center, Rochester, NY 14623, USA; kathy_hoeger@urmc.rochester.edu; 4 Perelman and Claudia Cohen Center for Reproductive Medicine, Weill Cornell Medicine, New York, NY 10065, USA; sdspando@med.cornell.edu

**Keywords:** Mediterranean Diet, Dietary Approaches to Stop Hypertension, ovary, fertility, metabolism

## Abstract

The relationship between diet quality and ovarian morphology has biological plausibility yet remains unclear and was therefore evaluated. In a multicenter cross-sectional analysis, four dietary patterns were scored for 111 consecutive reproductive-aged women (18–45 years) using (1) Healthy Eating Index (HEI-2015); (2) alternative HEI-2010; (3) alternate Mediterranean Diet (aMED); (4) and Dietary Approaches to Stop Hypertension (DASH) indices. Ovarian volume (OV) and follicle number per ovary (FNPO) were evaluated on transvaginal ultrasonography. Relationships between dietary and ovarian morphology indices were evaluated by linear regression and mediation analyses. Associations between aMED and DASH scores and OV/FNPO were completely mediated by obesity, insulin resistance, and hyperandrogenism (All: *p* < 0.05), unlike direct associations (All: *p* ≥ 0.89). Namely, a 1-standard deviation [SD] increase in aMED score was associated with decreases in OV (0.09 SD; 0.4 mL) through reducing waist circumference. Likewise, a 1 SD increase in aMED and DASH score was associated with decreases in OV (0.07 SD; 0.3 mL) by reducing glucose response to a 75 g glucose tolerance test. A 1 SD increase in DASH score was associated with decreased FNPO (0.07 SD; 2 follicles) by reducing free androgen index (All: *p* < 0.05). Adherence to aMED and DASH eating plans was indirectly associated with significant improvements in ovarian form, providing novel mechanistic insights for future interventions about contributions of diet quality on ovarian function.

## 1. Introduction

Dietary patterns are practical nutritional tools that reflect an individual’s usual dietary behaviors. The use of dietary patterns reduces the risk for collinearity, synergistic, and interactive effects among single dietary factors, accounting for complex interactions among multiple dietary factors that may influence the states of health and disease [1,2,3,4]. Current evidence supports the relationships between dietary patterns and the development of chronic diseases, including type 2 diabetes, cardiovascular disease, and certain gynecological cancers in postmenopausal women [5,6,7,8]. However, the link between dietary patterns and health outcomes in reproductive-aged women is less clear [9,10,11,12,13]. Previous studies in reproductive-aged women have mainly focused on maternal dietary patterns and pregnancy outcomes, including pregnancy rate [10,11], spontaneous abortion [9], time to pregnancy [12], preterm birth [14], and birth weight [15], but have yielded less consistent results, likely owing to variability in defining and assessing dietary patterns and challenges in the reproducibility of data-driven dietary patterns [16,17,18]. The relationship between major *a-priori* diet quality indices and reproductive health outcomes at earlier stages of the reproductive span remains poorly elucidated. 

Understanding these relationships is critical since on average, reproductive-aged women in North America have low diet quality [19,20,21,22] and increased obesity rates [23], which were shown to increase the propensity for ovulatory dysfunction, hormonal dysregulation, and infertility [24,25,26]. Animal studies evaluating the impact of diet on ovarian function support a negative influence of poor diet quality, including high-fat [27,28,29,30]; low-protein [31]; and, Western-style diets (combined high-calorie and high-fat [32] and combined high-calorie, high-fat, and high-sucrose [33]) on ovarian follicle development and ovarian morphology, which is exacerbated by androgen exposure [32]. With respect to human studies, a systematic review including women of reproductive age evaluated the relationship between dietary nutrients, dietary patterns, and food groups with ovarian reserve biomarkers (e.g., antral follicle count, follicle-stimulating hormone [FSH], anti-müllerian hormone [AMH] concentrations) and reported no or modest associations of some single nutrients/foods (e.g., dietary fiber, soy products) with ovarian reserve. The authors could not draw any definitive conclusions about dietary patterns [34], in part due to a paucity of human research, a focus on single foods or nutrients *per se* vs. dietary patterns, and lack of controlling for the effect of other food components [34].

The relationship between dietary patterns and ovarian morphology has biological plausibility. The link between poor diet quality, obesity, and metabolic aberrations, including insulin resistance (IR) and hyperinsulinemia, is well established [28,29,30,31]. IR and hyperinsulinemia are known to contribute to a state of functional hyperandrogenism, with obesity having synergistic effects [35,36,37]. Namely, insulin can aggravate gonadotropin-mediated steroid synthesis by the ovarian theca cells; increase corticotropin-stimulated adrenal androgen production [38]; suppress the hepatic synthesis of sex-hormone binding globulin (SHBG), and ultimately contribute to increases in bioavailable androgens [36,38,39]. Subsequently, hyperandrogenism disrupts normal ovarian follicle development leading to untimely follicle maturation, follicle arrest, and ovarian dysmorphology, as evidenced by increased antral follicle count and ovarian size [35,36,39,40]. These relationships are consistent with the notion that the ovary serves as an integration site for metabolic signals that regulate follicle growth and steroid synthesis. These interconnections between diet, metabolic, and hormonal aberrations may reflect a link between poor diet quality and ovarian dysfunction; however, the presence and magnitude of this link are unknown.

Ultrasonography is a non-invasive and reproducible tool to diagnose and monitor ovulatory disorders. Previously, we have shown ovarian characteristics on ultrasonography reliably reflect ovarian function and the severity of reproductive disturbance in women with ovulatory disorders, including polycystic ovary syndrome (PCOS) [41,42,43,44,45,46,47]. Specifically, an increased number of small follicles (<10 mm in diameter) was associated with androgen excess, obesity, and IR [45], whereas the development of larger (≥10 mm) follicles was linked to improved insulin sensitivity and glucoregulatory status and ovulatory potential [48]. Together, ultrasonographic assessment of ovaries may represent a valuable means to monitor hormonal and metabolic alterations in women of reproductive age [43,49,50].

To our knowledge, no efforts have been made to characterize the relationship between diet quality and ovarian morphology in reproductive-aged women. We hypothesized a lower diet quality, characterized by a less favorable dietary pattern, would be associated with enlarged ovaries and increased follicle counts in women of reproductive age. Therefore, we evaluated the linear associations between major *a-priori* dietary patterns and ovarian morphology indices as our primary objective. In addition to previous studies that have only examined linear associations between diet quality and reproductive health outcomes, we examined whether associations between diet quality and ovarian morphology were mediated by intermediate metabolic, endocrine, and/or clinical markers as our secondary objective. Mediation analysis allows for the exploration of associations between exposure variables and biological health outcomes that may not be captured by direct associations [51,52], in part, due to complex relationships between variables, such as diet and reproductive health outcomes. Therefore, in the present work, we examined whether the associations between diet and ovarian characteristics were direct or mediated by intermediate biological variable(s). Findings from the present study may provide deeper insights to elucidate the link between diet and female reproductive health.

## 2. Materials and Methods

### 2.1. Study Design and Setting

The present study represents a cross-sectional, secondary analysis of 122 consecutive women recruited to 4 ongoing studies (ClinicalTrials.gov Identifiers: NCT01859663, NCT01927471, NCT01927432, NCT01785719) between January 2013 and July 2018 that met the inclusion criteria detailed below. Recruitment was completed using paper and electronic advertisements at 3 academic institutions in New York State: (1) Division of Nutritional Sciences, Cornell University (Ithaca); (2) Department of Obstetrics and Gynecology, University of Rochester Medical Center (Rochester); and, (3) Ronald O. Perelman and Claudia Cohen Center for Reproductive Medicine, Weill Cornell Medicine (New York).

### 2.2. Ethical Approval 

The Institutional Review Boards at Cornell University, University of Rochester, and Weill Cornell Medicine approved the study protocols. All procedures were conducted in compliance with the World Medical Association Declaration of Helsinki, and the Guidelines of the International Conference on Harmonization on Good Clinical Practice. All participants provided written informed consent.

### 2.3. Study Participants

Women were eligible to participate if they were 18–45 years old and exhibited no signs of perimenopause. Exclusion criteria included: (1) the use of insulin-sensitizing medications within 2 months of study participation; (2) the presence of diabetes mellitus, hyperprolactinemia, untreated thyroid dysfunction, congenital adrenal hyperplasia or premature ovarian failure; and, (3) failure to visualize the ovaries on transvaginal ultrasonography. One hundred and twenty-two women provided complete ultrasonographic and dietary datasets. Eleven women were excluded from the present analysis due to implausible self-reported energy intakes of 96 and 560 kcal/d (*n* = 2), poor visualization of ovaries (*n* = 2), and use of hormonal contraception (*n* = 7). The final cohort included 111 women. 

### 2.4. Study Procedures 

#### 2.4.1. Clinical Assessments

A standardized health history and physical examination was performed to obtain demographics, anthropometry, vitals, and family history of chronic disease as described in greater detail previously [53]. Hirsutism was evaluated by visual and self-reported scoring of terminal hair growth on 9 regions of the body [54]. Clinical evidence of hyperandrogenism was defined by a modified hirsutism score ≥6 [55]. Menstrual cycle length was recorded as the self-reported average interval between menstrual cycles in the 12 months before study enrollment. Evidence of oligo-amenorrhea was defined by a self-reported average menstrual cycle length ≥36 days. Further, we determined the PCOS status of our entire cohort using the recommended thresholds of the 2018 International Evidence-based Guidelines for the Assessment and Management of PCOS [55] in compliance with the National Institutes of Health (NIH) criteria [56] to account for any impact of PCOS status on outcomes through sub-group analyses.

#### 2.4.2. Biochemical Analyses

Fasted blood samples were collected to measure biochemical markers, including insulin, glucose, total testosterone (TT), estradiol, and SHBG. Blood samples were also taken as part of assessing insulin and glucose responses to a standard 75 g oral glucose tolerance test (OGTT) at 30, 60, 90, and 120 minutes. Blood samples were collected during the early follicular phase in women with regular menstrual cycles or the absence of a dominant follicle (≥10 mm) or corpus luteum in those with irregular or absent menstrual cycles. Serum concentrations of insulin, estradiol, and SHBG were measured by chemiluminescence immunoassay (Siemens Medical Solutions Diagnostics, Deerfield, IL, USA) and glucose by commercial glucometer test strips (Accu-Chek Aviva Plus, Roche Diagnostics, Indianapolis, IN, USA). Serum concentrations of TT were measured by liquid chromatography-tandem mass spectrometry at a clinical chemistry lab participating in the Centers for Disease Control and Prevention Hormone Standardization Program (Brigham Research Assay Core, Boston, MA, USA), as previously described [53]. The homeostasis model assessment of insulin resistance (HOMA-IR) was calculated using the formula: fasting glucose (mg/dL) × fasting insulin (μIU/mL)/405 [57]. The area under the 2-hr response curves (AUC) for a 75-g OGTT for insulin and glucose was determined using the trapezoidal rule [58,59]. The free androgen index (FAI) was calculated using the formula: TT (nmol/L)/SHBG (nmol/L) × 100 [60]. Biochemical evidence of hyperandrogenism was defined by a fasting serum TT concentration ≥1.7 nmoL/L or FAI ≥6; thresholds reflected the 95th percentiles of androgen concentrations in an internal reference cohort. All samples were processed for serum and stored at −80 °C until the time of analyses. All inter- and intra-assay coefficients of variation were ≤10.5%, consistent with good assay performance.

#### 2.4.3. Ultrasonographic Assessments of Ovarian morphology

Transvaginal ultrasound scans were conducted at all study sites using a standardized protocol. Whole ovaries were scanned from their inner to outer margins in the longitudinal plane using a GE Voluson ultrasound machine (E8, S6, or S8 Series; GE Healthcare, Milwaukee, WI, USA) with a 5–9 or 6–12 MHz multifrequency transducer. Ultrasound examinations were conducted in the early follicular phase in women with regular menstrual cycles or the absence of a dominant follicle (≥10 mm) or corpus luteum in those with irregular or absent menstrual cycles. Two-dimensional cineloops in the longitudinal plane were archived for offline analysis using DICOM imaging software (Sante DICOM Editor, Santesoft LTD, Athens, Greece). Cineloops were reviewed by 1 of 6 investigators, who had each demonstrated strong inter-rater agreement in follicle counts as part of an internal reliability assessment (intraclass correlation coefficients >0.80). Endpoints of interest were (1) the total number of follicles per ovary (FNPO 2–9 mm); and, (2) mean ovarian volume (OV). Reliable follicle counts were obtained using the grid system [42]. OV was estimated in the largest cross-section of each ovary using the simplified formula for a prolate ellipsoid [61]. Mean follicle number per single cross-section (FNPS) ≥9 was used to identify polycystic ovaries if suboptimal image quality prevented a reliable evaluation of FNPO and/or OV [41].

#### 2.4.4. Dietary Assessments

Food consumption data was collected using VioScreen™ (Version 2.17; VioCare, Inc., Princeton, NJ). VioScreen is an adult-validated, self-administered web-based food frequency questionnaire (FFQ) [62]. Vioscreen FFQ has been used in both research and clinical settings to assess habitual diet over the past 3 months and uses graphics with approximately 1200 food images and branching questions that reduce missing foods and respondent burden [62,63]. Further details about the Vioscreen FFQ have been published [64]. Specific nutrient intakes and food data were calculated by processing the FFQ data using the Nutrition Data System for Research software (Version 42; Nutrition Coordinating Center, University of Minnesota, Minneapolis, MN, USA). All women responded to the FFQ in a clinical setting.

Four major *a-priori* dietary patterns were evaluated in the present study: (1) Healthy Eating Index-2015 (HEI-2015) [65,66,67]; (2) alternative Healthy Eating Index-2010 (aHEI-2010) [68]; (3) alternate Mediterranean Diet score (aMED) [7]; and, (4) and Dietary Approaches to Stop Hypertension (DASH) [69] based on the dietary information obtained from the FFQ. All dietary patterns derived from this FFQ were established *a-priori.* For each pattern, a higher score represented a healthier diet. The methods of scoring each dietary pattern are summarized below. 

Calculation of HEI-2015 was based on the United States Department of Agriculture (USDA) (Washington, DC, USA) and the National Cancer Institute (NCI), and aligned with the 2015–2020 Dietary Guidelines for Americans [70]. Briefly, the HEI-2015 consists of 13 food items. The first 6 items include (1) total vegetables; (2) total fruits; (3) whole fruits; (4) greens and beans; (5) seafood and plant proteins; and (6) total proteins, which can be scored from 0 to 5 points each. The remaining 7 items include (7) whole grains; (8) low-fat dairy; (9) fatty acids ratio (polyunsaturated fatty acids plus monounsaturated fatty acids to saturated fatty acids); (10) refined grains; (11) sodium; (12) added sugars; and (13) saturated fats, which can be scored from 0 to 10 points each. Most food components (except for fatty acids ratio, added sugars, and saturated fats) are scored on a density basis per 1000 kcal or as a percentage of energy. Four components (sodium, refined grains, added sugars, and saturated fats) are reverse scored (i.e., higher intakes receive lower scores). Total HEI-2015 scores were computed by aggregating scores across individual dietary components such that total scores ranged from zero (poor diet quality) to 100 (optimal diet quality) [65,66,71].

The aHEI-2010 index [68] is a modification to the USDA and NCI HEI-2010 dietary pattern index [72] and includes food items thought to influence the risk of chronic disease. The aHEI-2010 addresses quality within food groups, has predicted adherence and nonadherence criteria for each food group, and consists of 11 food items, of which, higher intakes are awarded for 6 components: (1) vegetables (excluding potatoes); (2) whole fruits; (3) whole grains; (4) nuts and legumes; (5) long-chain ω-3 polyunsaturated fatty acids; (6) polyunsaturated fatty acids (excluding long-chain ω-3 polyunsaturated fatty acids); moderate intake is awarded for (7) alcohol (0.5–1.5 drinks/d for women); and lower intakes are awarded for the 4 remaining components: (8) sugar-sweetened beverages and fruit juice; (9) red and processed meats; (10) sodium; and, (11) *trans* fatty acids. Each component intake is scored from 0 to 10. The score for each component was summed. The overall aHEI-2010 scores range from 0 (poor diet quality) to 110 (optimal diet quality) [68].

The aMED score is an adapted version of the traditional Mediterranean Diet score, which demonstrated favorable effects on the risk of chronic disease [73,74,75]. The aMED score was determined based on the intakes of 9 food items: (1) fruits; (2) vegetables (excluding potato); (3) nuts; (4) legumes; (5) whole grains; (6) fish; (7) ratio of monounsaturated to saturated fat; (8) red and processed meats; and, (9) alcohol. Those whose intake was above the median for fruits, vegetables, nuts, legumes, whole grains, fish, or a ratio of monounsaturated to saturated fat received 1 point for each category. Consumption of red and processed meat below the median was awarded 1 point, and alcohol intake 5–15 g/d was awarded 1 point; otherwise, zero points were received. The score for each component was summed. The total score range for the aMED was 0 (poor diet quality) to 9 (optimal diet quality) [7].

The DASH score was developed based on emphasized or minimized foods, nutrients, and beverages typically consumed in the DASH diet, which has been shown to reduce the risk of blood pressure in previous randomized controlled trials [76,77]. The DASH diet score consists of 8 food items: (1) fruits; (2) vegetables, (3) nuts and legumes, (4) low-fat dairy, (5) whole grains, (6) sodium, (7) sugar-sweetened beverages, and, (8) red and processed meats. The scoring was based on quintile rankings within the population studied. For fruits, vegetables, nuts and legumes, low-fat dairy, and whole grains, those in the highest quintile were given a score of 5; 1 point was subtracted for each decreasing quintile, and those in the lowest quintile receive a score of 1. For sodium, sweetened beverages, and red and processed meats, scoring was reversed: those in the highest quintile received a minimum score of 1, whereas participants in the lowest quintile were awarded a maximum score of 5. The score for each component was summed. The total DASH scores range from 8 (poor diet quality) to 40 points (optimal diet quality) [69].

#### 2.4.5. Statistical Analyses

Statistical analyses were performed using SPSS (Version 25.0, IBM, Armonk, NY, USA). Descriptive statistics were used to examine the characteristics of the study participants. Results were presented as mean (standard deviation [SD]) or number (percentage) for each group. Correlations between dietary patterns were assessed using the Spearman correlations. 

**Figure 1:**
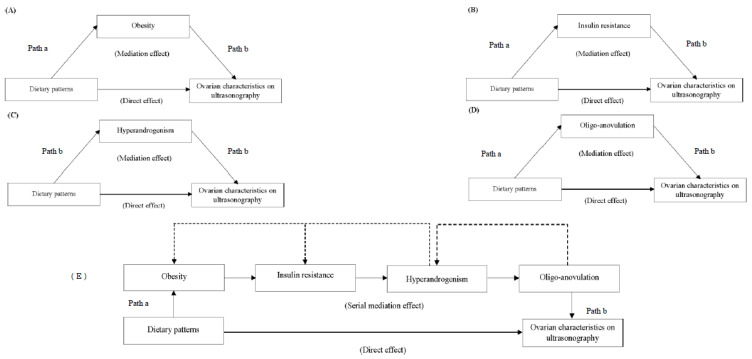
Regression modeling for direct and indirect mediation frameworks. The effect estimates of dietary patterns on ovarian morphology without taking into account mediators is known as “direct effect.” The effect estimates of dietary patterns on ovarian morphology while controlling for an individual mediator were fitted (**Panels A to D**) to illustrate “simple mediation.” The “serial mediation model” fitted the association between dietary patterns and ovarian morphology while accounting for all mediators (**Panel E**). The sum of direct and indirect effect estimates is known as “total effect.” The solid lines were accounted for in the pathway analyses. The dotted lines illustrate the pathophysiologic associations between mediators and were not included in the pathway analyses as per the objective of the study. Abbreviations: X, exposure; M, mediator; and Y, outcome variables.

We used mediation analysis in the present work. Mediation analysis hypothesized that the association between exposure and outcome is mediated by a mechanism factor: the mediator. To be considered a mediator, the following criteria needed to be satisfied: (1) a change in amounts of the exposure significantly affects the changes in the mediator (path a is significant) and (2) there is a significant relationship between the mediator and the outcome (path b is significant), and (3) there is a biological plausibility to support a relationship among exposure, mediator, and outcomes. Our conceptual framework based on the available evidence (Figure 1) and justifies exploratory hypothesis testing by mediation analyses. In contrast to the traditional logic of Baron and Kennedy in 1986 [78], a reduction of the effect of the exposure on the outcome in the presence of the mediator (the direct effect is significant) was not necessarily an *a-priori* assumption to conduct mediation analyses in our work. Rather, our approach was consistent with the recent advancements of Zhao et al. [79], Hayes and Rockwood [80] and MacKinnon et al. [81], which support the justification of mediation analysis where the null hypothesis (the indirect effect is zero) is rejected after a significant relationship between (1) the exposure and outcome and (2) the mediator and the outcome is observed. That is, the interpretation of mediation is measured by the size of the indirect effect, not by the lack of a direct effect *per se*. Moreover, this approach is more likely biologically and clinically translatable than traditional analyses to explain the relevance of nutrition to reproductive health, which is not always explained by simple associations [52].

All exposure, mediator, and outcome variables were examined for normality of residuals using linear regression analyses and visual inspection of histograms and probability plots. Based on the residual diagnostic results, all analyses were performed using log-transformed mediators and outcome variables. 

In the first stage of the analysis, linear regressions were performed to examine a “direct” link between dietary patterns and ovarian morphology, and to identify potential mediators that could explain an “indirect” effect between factors. Associations were examined between (1) dietary patterns and ovarian morphology; (2) dietary patterns and mediators; and (3) mediators and ovarian morphology, and unstandardized β was reported. Standardized β was also reported so that the effect estimates can be appropriately compared across the 4 evaluated dietary patterns.

In the second stage of the analysis, regression-based simple mediation tests were used to examine whether the associations between dietary patterns and ovarian morphology could be explained through indirect effects (also known as mediation effects) by each of the mediator variables. The regression-based simple mediation framework is illustrated in Figure 1A–D.

In the third stage of the analysis, serial multiple mediation regressions were used to examine the effects of dietary patterns on ovarian morphology through the pathways of a series of mediators. The regression-based serial mediation framework is illustrated in Figure 1E. 

Accordingly, β was estimated as the product-of-coefficient approach (unstandardized and standardized) at both (simple and serial) stages of mediation analyses. Details of interpretation of β at each stage are described in the Supplementary Data 1, Supplementary Data 2 (Table footnotes), and Figure legends, where appropriate. 

All the mediation analyses were adjusted for relevant lifestyle confounders, including age (y), race (categorical), and total daily energy intake (kcal/d). Given the potential mediating effect of obesity in the relationship between dietary patterns and ovarian morphology, we chose to explore the effects of BMI as a mediator factor and removed it from the covariate list. Because HEI-2015 and DASH scores do not include a specific component for alcohol, we ran additional sub-analyses adjusting for alcohol intake; however, since 51.4% (57/111) of our participants had 0–1.5 g/d alcohol intake, we chose to remove alcohol from the final adjustment. All statistical tests were based on *a-priori* hypotheses. Therefore, no adjustments were performed for multiple testing. 

The pathway analyses were performed using the PROCESS macro for SPSS (Version 3.4) developed by Hayes [82]. Our analyses drew 50,000 bootstrapped samples to estimate the most accurate effect estimate as small sample sizes may bias the evaluation of the mediation effect. Results were considered significant at *p* < 0.05.

## 3. Results

### 3.1. Descriptive Statistics of Participants

The characteristics of the study participants are presented in Table 1. Overall, women were young (mean age = 27.8 years) and primarily White (73/111 [65.8%]). Sixteen percent (18/111) of all women had obesity (BMI ≥ 30 kg/m^2^). Collectively, women had an intermediate diet quality, as evidenced by their dietary pattern scores (mean [SD], 65.5 [13] vs. total score range references, 1–100) for HEI-2015 score; (53.7 [13] vs. total score range references, 1–110) for aHEI-2010 score; (4 [2] vs. total score range references, 1–9) for aMED score; and, (24 [5] vs. total score range references, 8–40) for DASH score (Table 1). 

### 3.2. Correlations between Dietary Patterns

There were moderate correlations between the four evaluated dietary patterns (ρ = 0.48–0.73; *p* < 0.01; Table 2). The strongest correlations were observed between the aMED and DASH dietary indices (ρ = 0.73), and between the HEI-2015 and aHEI-2010 indices (ρ = 0.70). None of the evaluated dietary patterns were perfectly (ρ > 0.90) correlated to suggest multicollinearity (All: *p* < 0.01).

### 3.3. Direct Associations

#### 3.3.1. Direct Associations between Dietary Patterns and Ovarian Characteristics

Results of linear regression analyses showed no direct associations between ovarian characteristics and dietary patterns (All: *p* ≥ 0.89; Table 3). 

#### 3.3.2. Associations between Dietary Patterns and Metabolic and Reproductive Measures (Path a)

There were inverse associations between the markers of obesity, including BMI, WC, and WHR, and all four dietary patterns (Supplementary Table S1). A 1-SD increase in the aMED diet score was associated with a 0.26 SD lower BMI (*p* < 0.05) and 0.28 SD lower WC (*p* < 0.01). Increased HEI-2015, aHEI-2010, and aMED scores were associated with reduced fasting insulin, HOMA-IR index, and glucose AUC (*p* < 0.05, Supplementary Table S1). Namely, a 1-SD increase in the aMED diet score was associated with a 0.25 SD lower fasting insulin, 0.23 SD lower HOMA-IR, and 0.22 SD lower glucose AUC (All: *p* < 0.05). Similarly, there were negative associations between FAI and HEI-2015, aHEI-2010, and DASH scores (All: *p* < 0.05). A 1-SD increase in the DASH score was associated with 0.25 lower FAI (*p* < 0.05; Supplementary Table S1). 

#### 3.3.3. Associations between Metabolic and Reproductive Profiles and Ovarian Characteristics (Path b)

There were associations between BMI, WC, and OV (Supplementary Table S2). Specifically, a 1-SD increase in BMI and WC was associated with 0.24 and 0.21 SD increase in OV, respectively (*p* < 0.05). Similarly, there were associations between these ovarian characteristics and hyperandrogenism and menstrual cycle length. Specifically, a 1-SD increase in TT concentrations and length of the menstrual cycle was associated with a 0.25 and 0.50 SD increase in FNPO, respectively (*p* < 0.05; Supplementary Table S2).

### 3.4. Indirect Associations

#### 3.4.1. Simple Mediation Analyses Evaluating Associations between Dietary Patterns and Ovarian Characteristics

**Figure 2:**
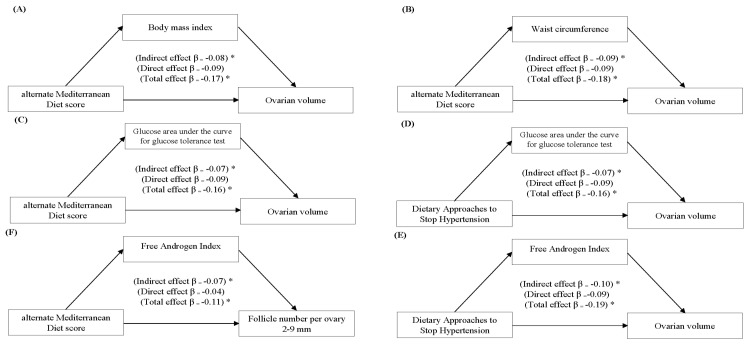
Simple mediation analyses demonstrating indirect associations between aMED and DASH scores and ovarian characteristics on ultrasonography for *n* = 109 women who provided optimal image quality with a reliable evaluation of ovarian volume (**Panels A to E**) and follicle numbers per ovary (**Panel F**). β is a standardized product of serial regression models on log-transformed variables. Standardized β can be interpreted as the expected standard deviation (SD) change in the OV and FNPO per 1 SD increase in dietary patterns through an individual mediator. Body mass index and waist circumference were designated as a proxy for obesity; glucose area under the 75 g oral glucose tolerance test curve for insulin resistance; and, free androgen index for hyperandrogenism based on the magnitude of the effect estimates in our statistical models shown in Supplementary Data 2 (Supplementary Tables S1 and S2). All models are adjusted for age, race, and total energy intake. An asterisk above the effect estimate denotes a significant effect estimate (*p* < 0.05).

Results of simple mediation analyses revealed that BMI, WC, glucose AUC, and FAI individually mediated the relationship between aMED and DASH dietary patterns and the markers of ovarian characteristics (Figure 2). After adjusting for age, race, and energy intake, a 1-SD increase in the aMED score was associated with 0.08 (Figure 2A) and 0.09 (Figure 2B) SD decrease in OV through reduced BMI and WC, respectively (*p* < 0.05). A 1-SD increase in the aMED (Figure 2C) and DASH (Figure 2D) scores decreased OV by 0.07 SD through decreased glucose AUC (*p* < 0.05). A 1-SD increase in the DASH score was associated with a 0.10 (Figure 2E) and 0.07 (Figure 2F) SD decrease in OV and FNPO, respectively, through reduced FAI (*p* < 0.05). More details of simple associations across all evaluated dietary patterns are available in Supplementary Table S3. 

#### 3.4.2. Serial Mediation Analyses Evaluating Associations between Dietary Patterns and Ovarian Characteristics

**Figure 3:**
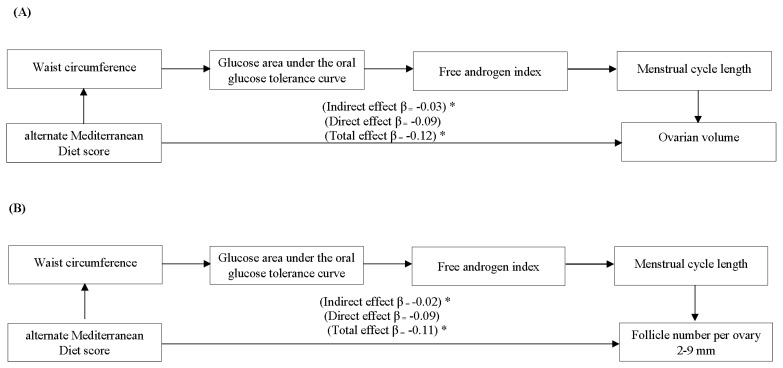
Serial mediation analyses demonstrating indirect associations between aMED score and ovarian characteristics on ultrasonography for *n* = 109 women who provided optimal image quality with a reliable evaluation of ovarian volume (**Panel A**) and follicle number per ovary (**Panel B**). β is a standardized product of serial regression models on log-transformed variables. Standardized β can be interpreted as the expected standard deviation (SD) change in the OV and FNPO per 1 SD increase in dietary patterns through serial mediators. Waist circumference was selected as a proxy for obesity; total glucose area under the 75 g oral glucose tolerance test curve for insulin resistance; free androgen index for hyperandrogenism; and menstrual cycle length for oligo-anovulation based on the magnitude of the effect estimates demonstrated in our statistical models shown in Supplementary Data 2 (Supplementary Tables S1 and S2). All models are adjusted for age, race, and total energy intake. An asterisk above the effect estimate denotes a significant effect estimate (*p* < 0.05).

Results of serial mediation analyses showed indirect associations between aMED and ovarian characteristics after adjusting for age, race, and energy intake (Figure 3). The associations were sequentially mediated by WC, glucose AUC, FAI, and length of the menstrual cycle. A 1-SD increase in the aMED score was associated with a 0.03 SD decrease in OV (Figure 3A) and a 0.02 SD decrease in FNPO (Figure 3B). More details of serial associations across all four dietary patterns are available in Supplementary Table S4. Indirect associations between dietary patterns and ovarian characteristics yielded smaller effect sizes that did not remain significant across smaller PCOS and control subgroups (All *p* > 0.05; data not shown) yet showed the comparable direction of associations in each subset when compared to all women.

## 4. Discussion

To our knowledge, this is the first study to evaluate the relationship between diet quality and ovarian morphology. While we observed no direct associations between diet quality, as assessed by dietary patterns, and ovarian morphology on ultrasonography, our findings suggest that adherence to the Mediterranean diet and DASH eating plan are indirectly associated with improved ovarian morphology, as evidenced by lower follicle populations and ovarian size. Our observations revealed that obesity, as evaluated by increased BMI and WC, IR as evaluated by increased glucose response to OGTT, and hyperandrogenism, as evaluated by increased FAI, are likely the key factors in explaining the indirect relationship between lower diet quality and ovarian dysmorphology. Our observations are hypothesis-generating only and provide a novel framework for future intervention studies to elucidate the clinical significance of these associations on ovarian function and the underlying mechanisms through which diet could affect ovarian function across a spectrum of reproductive potential. 

We observed that adherence to aMED and the DASH eating plan was indirectly associated with decreased FNPO and OV. These observations are consistent with a higher likelihood of normal ovarian form and function in women with improved hormonal and/or metabolic status that consume healthier diets [41,45,46,83,84,85]. Both increased follicle counts and enlarged ovaries are recognized risk determinants for chronic anovulation and subfertility [49]. Accumulation of small follicles reflects impairments in the follicle development, which are known to exacerbate anovulation [24,50,86,87] through pre-established mechanisms [24,40,88,89]. Likewise, enlarged ovaries may be the result of hyperplasia in ovarian connective tissue associated with excessive androgen synthesis and anovulation [37,90,91]. 

The correlations among the evaluated dietary indices were moderate (ρ = 0.48–0.73) since all indices were designed to emphasize higher consumption of fruits, vegetables, and whole grains and de-emphasize the consumption of red and processed meat. By contrast, each dietary pattern represented a unique combination of dietary components, scoring system, and classification methodology; thereby, individual evaluations were feasible [7,65,66,68,69,71]. There are important differences between aMED and DASH scores and evaluated HEI indices in the present study that can explain our observations [92]. Both the aMED and DASH scores were designed to emphasize the intake of foods versus nutrients and have fewer food items (8 and 9, respectively). However, only the aMED score has individual components for legumes, nuts, and fish. Consumption of these food items has been emphasized in the aMED score more than the other indices. Moreover, aMED includes the ratio of monounsaturated to saturated fat. On the other hand, only the DASH diet has sodium, low-fat dairy, and sugar-sweetened beverage components compared to other indices. 

Mechanisms through which specific dietary patterns can affect ovarian morphology appear to majorly target adiposity and metabolic status. The aMED diet and DASH eating plan are characterized by favorable combinations of dietary components, including high dietary fiber, refined composition, and proportion of macronutrients, vitamins, minerals, and other dietary factors with antioxidant properties, such as phenolic compounds (e.g., resveratrol) derived from complex carbohydrates [69,75,93,94,95]. Refinement in the composition and dosage of dietary carbohydrate and fiber and anti-inflammatory/oxidative properties of these diets have been shown to decrease IR, dysglycemia, hyperandrogenism, obesity, and induce satiety and weight loss in women with anovulation and abnormal ovarian morphology, such as PCOS in recent years [22,85,96,97,98,99,100,101,102,103] and their respective mechanisms have been described in greater detail previously [94,104,105,106,107,108,109,110,111,112,113,114]. Further, the DASH eating plan includes a low saturated fat dairy component that provides vitamin D, whereas aMED includes a fish component rich in ω-3 polyunsaturated fat and is designed to increase the ratio of monounsaturated fat to saturated fat intake. These refinements in the type and composition of fatty acid intakes have been shown to improve ovarian steroidogenesis [115] and influence follicular development by modulating serum FSH concentrations in women [116,117]. A recent report of negative associations between serum AMH concentrations with mono- and polyunsaturated fatty acids in healthy women [118] supports the protective effects of these fatty acids on follicular development. Vitamin D receptors are widely distributed across the female reproductive system, including the ovaries [119]. Animal studies have shown the benefits of vitamin D intake on ovarian steroidogenesis and follicular maturation [120,121]. However, the influence of vitamin D intake on female reproductive health remains unknown [122,123,124]. The aMED and DASH indices emphasize reducing the intake of red meat and increasing protein intake from fish and dairy products. Previous studies have shown the benefits of dairy protein on decreasing antral follicle counts in infertile women [125] and fish consumption on blastocyst formation [126], as well as the adverse effects of red meat consumption on ovulation [127]. Decreased consumption of sugar-sweetened beverages and dietary sodium is recommended in the DASH index and was shown to increase glucose regulation [128], reduce IR [129], and abdominal adiposity [130] in women. Together, our observations support emerging evidence on the benefits of adherence to aMED and DASH eating plans on improving obesity and metabolic control in women with ovarian dysfunction, including PCOS [94,101,131,132,133]. These observations may also suggest a potential benefit of these dietary patterns on ovarian morphology, which has implications for the clinical management of women with ovulatory disorders. However, more research is required to confirm these findings and identify any potential direct effects of diet on ovarian health. 

Our conclusions are subject to limitations inherent to the cross-sectional design, small sample size, and incomplete knowledge of all potential covariates and other influencing factors, including physical activity, smoking, infertility or pregnancy history, which likely affect female reproductive health [134]. Assessment of oocyte quality was beyond the scope of the present work. Our study included both women with and without PCOS. Our subgroup analyses revealed similar patterns/directions of associations between diet quality and ovarian morphology in PCOS and control subsets when compared to all women, yet marginally smaller effect sizes that did not reach statistical significance possibly due to our smaller sample sizes in each sub-set. However, we acknowledge that defining women based on the NIH criteria does not account for polycystic ovaries, thereby neglecting milder phenotypes of PCOS. We were restricted in expanding on our mediation models and to model combined serial and parallel mediation and moderation processes by including additional biological variables (e.g., estrogen status) to test our hypothesis based on *a-priori* variables (e.g., obesity, insulin, and androgen status) due to the PROCESS macro limitations, which are not uncommon in studies of this type [135]. Therefore, the present work does not account for any complex (synergistic or antagonistic) interactions among mediators. Subjective assessments of dietary intake may tend toward random or systematic error, recall bias, underreporting, and reactivity [136,137]. This study also has several strengths. We used a multi-dimensional and comprehensive approach to identify the relationship between diet quality and ovarian morphology [6,67] and evaluated diet quality using major *a-priori* established dietary patterns. We uniformly evaluated ovarian morphology using high-resolution real-time ultrasonography and meticulous *post hoc* analysis of FNPO [42,138] and used a gold standard methodology for measuring TT concentrations. Our assessment of ovarian morphology was based on a single cycle vs. serial assessments, which limits our ability to detect inter-cycle variability in ovarian morphology that has been shown to be moderate in some studies [139,140,141,142,143]. Therefore, future research is required to evaluate longitudinal changes in ovarian morphology in response to adherence to aMED and DASH eating plans to provide deeper insight into the potential relationship between diet and ovarian function and reveal a more comprehensive and reliable picture about when these changes may become evident. Nevertheless, our findings are also particularly informative since adherence to the Mediterranean and DASH dietary patterns have been implicated in reducing the risk of chronic disease and certain gynecologic cancers [2,69,75,76,93,95,144,145,146] and could be used to improve reproductive and metabolic health in women with ovarian dysfunction, including PCOS [101,132].

## 5. Conclusions

Women of reproductive age who consume a healthy diet consistent with the Mediterranean and DASH eating patterns may have improved ovarian morphology that likely reflects an improved ovarian function. The association between diet quality and ovarian morphology was primarily mediated by decreases in weight, IR, and hyperandrogenism. Our findings are consistent with emerging evidence about the benefits of these diets on obesity and the metabolic status of reproductive-aged women. Our findings add a new dimension to the existing evidence and provide a conceptual framework for future studies to understand the underlying mechanisms through which diet could affect ovarian health. By extrapolation, our findings have implications for future intervention studies to evaluate the impact of these dietary patterns on ovarian function in reproductive-aged women.

## Figures and Tables

**Table 1:** Descriptive characteristics of women (*n* = 111).

Measures (Measurement Unit)	Mean (SD) or Number (%)
Demographics	
Age (y)	27.8 (6.3)
Race (*n* [%])	
African American	13 (11.7)
Asian	12 (10.8)
White	73 (65.8)
Other	13 (11.7)
DM FHx (*n* [%])	63 (56.8)
CVD and/or HTN FHx (*n* [%])	72 (64.9)
PCOS Dx (*n* [%])	39 (35.1)
Dietary characteristics	
Total energy intake (kcal/day)	2237 (1000)
Dietary index	
HEI-2015 score	65.5 (13)
aHEI-2010 score	53.7 (13)
aMED score	4 (2)
DASH score	24 (5)
Alcohol intake (g/day)	8.9 (13)
Anthropometric characteristics	
BMI (kg/m^2^)	30.3 (8.1)
WC (cm)	91.9 (19.7)
WHR	0.84 (0.08)
Overweight (*n* [yes %])	56 (50.5)
Obesity (*n* [yes %])	18 (16.2)
Endocrine measures	
Fasting insulin (µIU)	12.3 (10.1)
FPG (mg/dL)	5.1 (0.5)
HOMA-IR index (%)	2.6 (2.4)
Insulin AUC (µIU/mL × 120 min)	8495 (6167)
Glucose AUC (mg/dL × 120 min)	756 (1.7)
TT (nmol/L)	1.7 (0.7)
Estradiol (pmol/L)	202.4 (120.4)
Modified hirsutism score	6 (5)
SHBG (nmol/mL)	49.8 (34.3)
FAI (%)	5 (4)
Menstruation history	
Menstrual cycle length (d)	65 (71)
Markers of ovarian morphology	
OV (mL)	9.6 (5.0)
FNPO 2–9 mm (*n*)	34 (23)

Abbreviations: DM2, type 2 diabetes; FHx, Family history diagnosis; CVD, cardiovascular disease; HTN, hypertension; PCOS, polycystic ovary syndrome; Dx, diagnosis; HEI-2015, Healthy Eating Index-2015; aHEI-2010, alternative Healthy Eating Index; aMED, alternate Mediterranean Diet score; DASH, Dietary Approaches to Stop Hypertension; BMI, body mass index; WC, waist circumference; WHR, waist to hip ratio; FPG, fasting plasma glucose; HOMA-IR, homeostatic model assessment of insulin resistance; AUC, area under the (75 g oral glucose tolerance test) curve; TT, total testosterone, SHBG, sex–hormone-binding globulin; FAI, free androgen index; OV, ovarian volume; FNPO, follicle number per ovary.

**Table 2:** Correlation between elevated dietary index scores for all women (*n* = 111).

Dietary Index	HEI-2015	aHEI-2010	aMED	DASH
HEI-2015	1.00 ^a^	0.70 ^a^	0.48 ^a^	0.53 ^a^
aHEI-2010		1.00 ^a^	0.63 ^a^	0.62 ^a^
aMED			1.00 ^a^	0.73 ^a^
DASH				1.00 ^a^

Abbreviations: HEI-2015, Healthy Eating Index-2015; aHEI-2010, alternative healthy eating index; aMED, alternate Mediterranean Diet score; DASH, Dietary Approaches to Stop Hypertension. a: Correlations were estimated using the Spearman (ρ) coefficients and were significant (*p* < 0.01).

**Table 3:** Direct associations between dietary patterns and ovarian morphology (*n* = 111).

Measures (Unit)	HEI-2015		aHEI-2010		aMED		DASH	
	Unstandard β_Direct_	Standard β_Direct_	Unstandard β_Direct_	Standard β_Direct_	Unstandard β_Direct_	Standard β_Direct_	Unstandard β_Direct_	Standard β_Direct_
OV (mL)	0.00	−0.06	0.00	−0.07	−0.02	−0.09	−0.01	−0.09
FNPO 2–9 mm (*n*)	0.00	−0.02	0.00	0.03	−0.01	−0.04	0.00	0.05

Abbreviations: HEI-2015, Healthy Eating Index-2015; aHEI-2010, alternative Healthy Eating Index; aMED, alternate Mediterranean Diet score; DASH, Dietary Approaches to Stop Hypertension; OV, ovarian volume; FNPO, follicle number per ovary. Unstandardized and standardized βDirect are slopes of linear regression models on log-transformed variables. Unstandardized βDirect can be interpreted as the expected change in the OV and FNPO per 1-unit increase in dietary patterns. Standardized βDirect can be interpreted as the expected standard deviation (SD) change in the OV and FNPO per 1-SD increase in dietary patterns (All: *p* ≥ 0.89).

## Supplementary Materials

The following are available online at https://www.mdpi.com/2072-6643/12/7/1953/s1, Supplementary Data 1: More Comprehensive Version of the Statistical Analyses; Supplementary Data 2: Table of Contents.
